# Genome-Wide Association Study of Root-Lesion Nematodes Pratylenchus Species and Crown Rot *Fusarium culmorum* in Bread Wheat

**DOI:** 10.3390/life12030372

**Published:** 2022-03-04

**Authors:** Quahir Sohail, Gul Erginbas-Orakci, Fatih Ozdemir, Abdulqader Jighly, Susanne Dreisigacker, Harun Bektas, Nevzat Birisik, Hakan Ozkan, Abdelfattah A. Dababat

**Affiliations:** 1 AgroBioSciences (AgBS), University Mohammed VI Polytechnic (UM6P), Ben Guerir 43150, Morocco; sohail.quahir@um6p.ma; 2 Global Wheat Program, International Maize and Wheat Improvement Center (CIMMYT), Ankara 06810, Turkey; g.erginbas@cgiar.org; 3 Bahri Dağdaş International Agricultural Research Institute, Konya 42020, Turkey; ozdemirfatih@tarimorman.gov.tr; 4 Agriculture Victoria, AgriBio, Centre for AgriBiosciences, Bundoora, VIC 3083, Australia; abdulqader.jighly@agriculture.vic.gov.au; 5 Global Wheat Program, International Maize and Wheat Improvement Center (CIMMYT), Texcoco CP56237, Mexico; s.dreisigacker@cgiar.org; 6 Department of Agricultural Biotechnology, Faculty of Agriculture, Siirt University, Siirt 56100, Turkey; harunbektas@siirt.edu.tr; 7 Ministry of Agriculture and Forestry (TAGEM), Ankara 06800, Turkey; nevzat.birisik@tarimorman.gov.tr; 8 Department of Field Crops, Faculty of Agriculture, Çukurova University, Adana 01330, Turkey

**Keywords:** Fusarium, genome-wide association, marker-trait association, Pratylenchus, wheat

## Abstract

*Triticum aestivum* L., also known as common wheat, is affected by many biotic stresses. Root diseases are the most difficult to tackle due to the complexity of phenotypic evaluation and the lack of resistant sources compared to other biotic stress factors. Soil-borne pathogens such as the root-lesion nematodes caused by the Pratylenchus species and crown rot caused by various Fusarium species are major wheat root diseases, causing substantial yield losses globally. A set of 189 advanced spring bread wheat lines obtained from the International Maize and Wheat Improvement Center (CIMMYT) were genotyped with 4056 single nucleotide polymorphisms (SNP) markers and screened for root-lesion nematodes and crown rot resistance. Population structure revealed that the genotypes could be divided into five subpopulations. Genome-Wide Association Studies were carried out for both resistances to Pratylenchus and Fusarium species. Based on our results, 11 different SNPs on chromosomes 1A, 1B, 2A, 3A, 4A, 5B, and 5D were significantly associated with root-lesion nematode resistance. Seven markers demonstrated association with *P. neglectus,* while the remaining four were linked to *P. thornei* resistance. In the case of crown rot, eight different markers on chromosomes 1A, 2B, 3A, 4B, 5B, and 7D were associated with Fusarium crown rot resistance. Identification and screening of root diseases is a challenging task; therefore, the newly identified resistant sources/genotypes could be exploited by breeders to be incorporated in breeding programs. The use of the identified markers in marker-assisted selection could enhance the selection process and cultivar development with root-lesion nematode and crown rot resistance.

## 1. Introduction

Bread wheat (*Triticum aestivum* L.) is one of the most important cereal crops for human consumption around the world. Wheat provides around 20% of human daily caloric intake. It is the main source of carbohydrates and is a significant source of proteins, inorganic ions, and vitamins [1]. Wheat is used to make flatbread, raised bread, cookies, cakes, porridges, noodles, muffins, pastries, sweets, pasta, etc.

Wheat production is limited by many abiotic and biotic stress factors. Among them, various wheat root diseases and pathogens cause significant yield losses [2,3]. Biotic stresses affecting the root system are extremely difficult to deal with, as phenotyping for resistance is a challenging task. Early detection of root diseases is a big challenge, and many farmers do not even know that their wheat fields are exposed to such diseases [3].

Based on economic impact, parasitic nematodes and Fusarium are among the main soil-borne pathogens attacking plant roots. The root-lesion nematodes (RLN) *Pratylenchus* sp. (*P. neglectus* and *P. thornei*) are plant-parasitic nematodes that feed on wheat roots [4,5]. These nematodes usually pierce the root cell walls, which consequently limits the nutrient and water uptake. This reduction in nutrient uptake, in turn, causes drought-like or nutrient deficiency symptoms, eventually resulting in yield loss and also damaging grain quality [6,7]. The two *Pratylenchus* species are the most common RLN species in wheat and usually coexist in fields [8]. They cause the most damage in temperate and dry regions of the U.S., Northern Canada, Latin America, Australia, the Middle East, etc. [9].

Another economically important wheat root disease is Fusarium crown rot (CR), which is caused by various Fusarium species [10,11,12]. The occurrence of this disease has become more common with the application of minimum tillage because Fusarium species survives on crop residue and infect following seasons wheat crop [13]. The Fusarium species initiates infection of wheat plants and colonizes the lower internodes causing root crowns, subsequently developing necrosis in the crowns in the lowest part of the stem and the root tissues resulting in the crown rot disease [14]. The range of options is quite limited to control CR and RLNs. Chemical control is somehow effective but neither economically feasible nor environmentally sustainable [6,7,15].

Identifying genetic resistance in wheat could provide a valuable alternative to the conventional control methods. Genetic approaches are potentially the most important strategies to effectively control CR and nematodes [4,16,17,18,19]. Genetic resistance is the best strategy of control, especially in developing countries where farmers have limited recourses to invest in crop protection [20]. However, detecting the disease occurrence, effective screening, and breeding for resistant genotypes is challenging. Bioassays providing reliable, accurate, and rapid screening results are quite limited, and screening facilities are not widely available. Large variation of the pathogen in the soil, evolving pathogen populations, and the environmental conditions that affect the disease make screening a big challenge [13]. The biggest problem, however, is the limited number of genetic resources that show high levels of resistance for both CR and RLN.

One way to screen large numbers of genetic resources is to take advantage of molecular markers. Marker-trait associations (MTAs) can be discovered using genome-wide association study (GWAS) [21,22]. In GWAS, a panel of genotypes is screened with a large number of genome-wide markers for detecting associations with important morphological, agronomic, or disease-resistance traits. An important advantage of GWAS is that it does not require the development of specific genetic mapping populations [23], and it can be conducted on breeding material or natural populations, allowing direct inference for data analysis to a breeding program. Therefore, in this study, we analyzed the association of single nucleotide polymorphism (SNP) markers with resistance responses of a panel of spring bread wheat accessions against RLN (*P. neglectus* and *P. thornei*) and CR (*F. culmorum*) diseases to identify novel sources of resistance to soil-borne diseases.

## 2. Results

### 2.1. Phenotypic Data Description for PT, PN, and CR

The results of the variance analysis (ANOVA) revealed significant differences among all experiments and growth conditions (Table 1). Accordingly, broad-sense heritability values of the means (H^2^) ranged between 0.79 (CR_Y) and 0.98 (PN2). The results suggest a high level of heritability for the evaluated traits in wheat.

**Figure 1:**
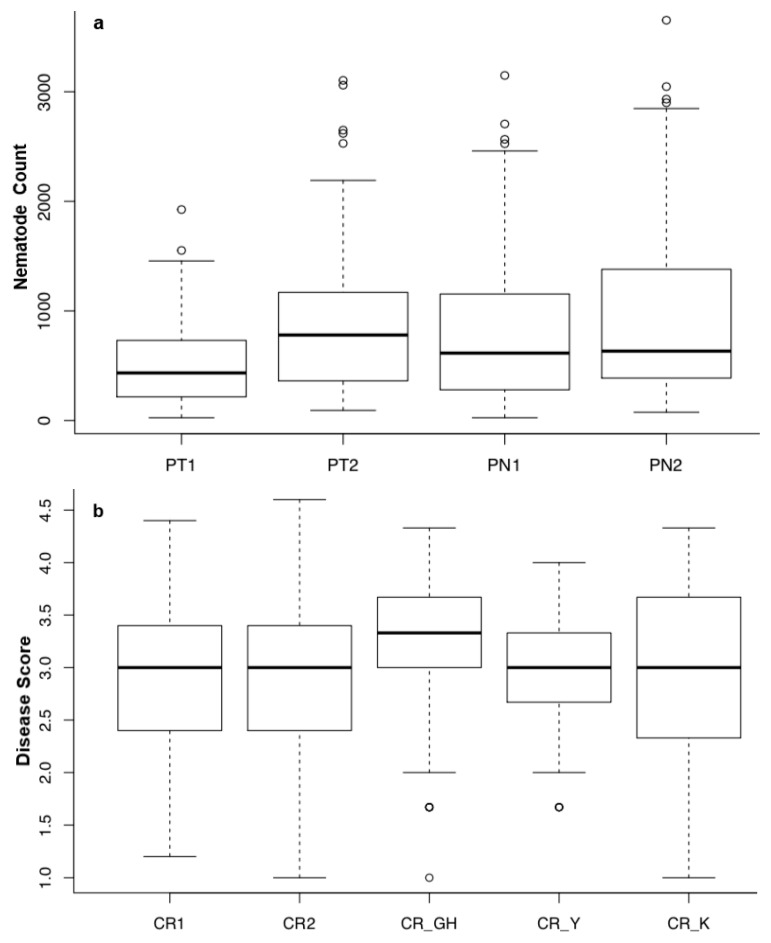
Ranges and the mean values across the spring bread wheat lines for disease scores against (**a**) *P. neglectus* (PN), and *P. thornei* (PT) in PN1-2 and PT1-2 experiments, and (**b**) crown rot (*Fusarium culmorum*) in growth room (CR1-2), greenhouse (GH), and two fields (CR_Y and CR_K) experiments. Plants were scored as the number of nematodes per plant in PN1-2 and PT1-2, while for crown rot, typical symptoms of browning percentage on the crown and the main stem were calculated following numeric scales (1–5) modified from Wildermuth et al. (1994): resistant (1: 1–9%), moderately resistant (2: 10–29%), moderately susceptible (3: 30–69%), susceptible (4: 70–89%), and highly susceptible (5: 90–99%).

Two consecutive GR experiments were conducted with *Pratylenchus* sp. (*P. neglectus* and *P. thornei*) for resistance assessment (Table S2 and Figure 1). The screening of wheat lines and checks revealed significant genetic variation in response to the nematode infection (Table S2). In the first experiment for *P. thornei* (PT1), the nematode count (nematodes/plant) ranged from 25 (GID6176558) to 1925 (GID6181747), with a mean value of 506.56 while in the second experiment (PT2), the range of nematodes/plant were 92 (GID6176334) to 3104 (GID6624543), and the mean value was 849.75. Among the checks, the cultivar ‘Seri’ had the highest nematode count (890 and 944) while the line Croc_1/*Ae.squarrosa*(224)//Opata had the lowest nematode count (247 and 264) in the PT1 and PT2 trials, respectively. The number of *P. neglectus* nematodes in PN1 and PN2 ranged from 25 (GID6176409) to 3149 (GID6417653) and 75 (GID6280393) to 3653 (GID6417653), respectively. The mean values for PN1 and PN2 were 789.95 and 948.05, respectively. The lines with the lowest PN count according to GR1 and GR2 mean values were GID6278849 (82.5 plant^−1^), GID6176409 (85 plant^−1^), and GID61781747 (90 plant^−1^). For the resistance against PT, the lines with the lowest mean nematode counts in GR1 and GR2 were GID6176334 (77 plant^−1^), GID6174858 (93.5 plant^−1^), and GID6174847 (97.5 plant^−1^). Overall means for PN were higher than for PT. Out of the 189 lines tested, 113 (59.8%) and 115 (60.8%) had nematode counts below average in PN1 and PN2, respectively. For the PT experiments, 104 (55%) and 105 (55.6%) lines had nematode counts below the average. The range for PN and PT counts is given in Figure 1, and the highest nematode count was obtained in the PN2 experiment (Figure 1). The check cultivar ‘Croc_1/*Ae.squarrosa*(224)//Opata had the lowest nematode count for both PN1-2 and PT1-2 experiments (Table S3). Accordingly, 57 (30%), 40 (21%), 42 (22%) and 51 (27%) lines had lower nematode counts in PT1, PT2, PN1 and PN2, respectively, compared to Croc_1/*Ae.squarrosa*(224)//Opata. Overall, out of the panel we evaluated for CR, PN, and PT resistance, six genotypes were resistant against CR, PT, and PN. Genotypes GID6181748, GID6278812, GID6279005, GID6424861, GID6424874 and GID6487731 showed moderate to complete resistance against crown-rot and root-lesion nematodes.

Crown rot scoring for seedling resistance was conducted in two growth room experiments (CR1 and CR2), and scores ranged from 1.2 (GID6175213) to 4.4 (GID6417653) in CR1 and between 1.1 (GID6279632) and 4.6 (GID6174901) in CR2 (Table S2 and Figure 1). The checks ‘2-49′ and ‘Suzen’ had the lowest scores, i.e., 2.2 for CR1 and 2.4 for CR2. In the CR_GH experiment, the CR score ranged from 1.0 to 4.3. In the field experiment in Yozgat (CR_Y) and Konya (CR_K), the CR scores range between 1.67 and 4.0 and 1.0 and 4.3, respectively. For CR_GH, the check genotype 2-49, Sunco and Suzen had the lowest score (2.3). Based on overall data, out of the 189 genotypes, around 31% were resistant to CR, 15% were moderately resistant, 15% were moderately susceptible, and 38% were very susceptible.

Correlation analysis was performed to observe similarities between GR, GH, and field experiments and between different seasons. Thus, we carried out a correlation analysis between CR, PN, and PT experiments (Table 2). Based on correlation analysis, the highest correlations were observed between PN1 and PN2 (0.9) and between CR1 and CR2 (0.72). There were also, moderate correlations between PT1 and PT2 (0.290), PN1 (0.309), PN2 (0.219); between PT2 and PN1 (0.341) and PN2 (0.323). On the other hand, significant negative correlations were observed between CR_K and PT2 (−0.213) and PN2 (−0.232). CR_K and CR_Y had significant positive correlations (0.532). According to correlation results, field values from Konya and Yozgat were in close association, and the results for PN and PT were all correlated. However, when field and controlled conditions (GR and GH) results were compared for CR, there was no clear correlation.

### 2.2. Genotypic Data and Population Structure

Genotyping-by-sequencing (GBS) yielded a total of 4056 SNP markers after initial filtering for missing data. The distribution of markers was the highest in the B genome with 2013 SNPs, followed by the A genome with 1518 SNPs and the D genome with 429 SNPs. The highest marker density was observed in B, and the lowest was observed in the D genomes, as expected in wheat. This is due to the recent addition of the D genome to the bread wheat genome compared to A and B genomes, and relatively lower marker information is available in the GBS databases. A total of 96 SNPs out of 4056 were not assigned into any genome, and they were classified as unknown. Of the 21 linkage groups, chromosome 2B had the highest number of markers (434 SNPs), while 4D had the least number of markers (17 SNPs).

**Figure 2:**
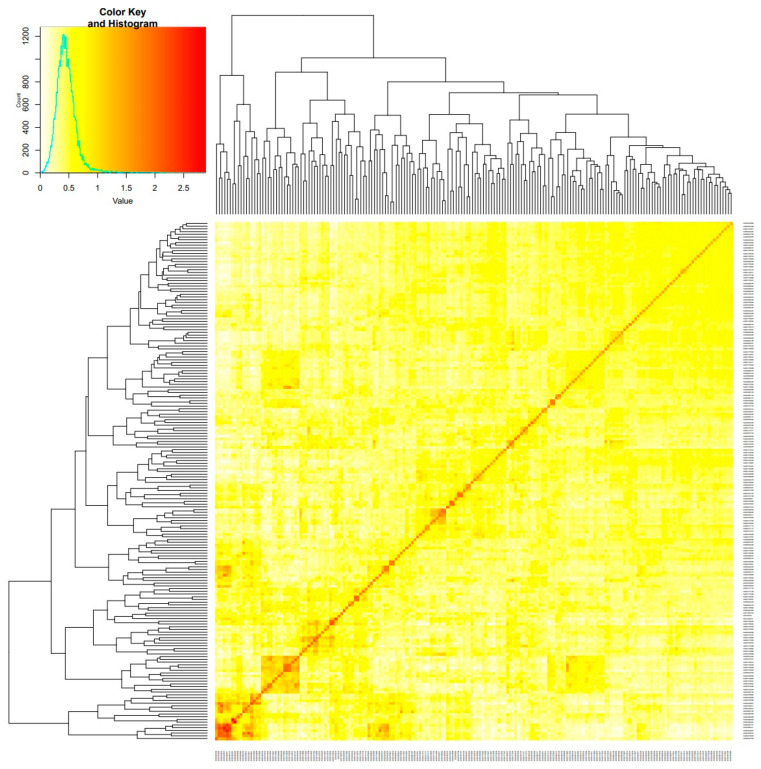
Heat map of kinship matrix using the IBS method among 189 spring wheat accessions genotyped with 4056 SNP markers.

**Figure 3:**
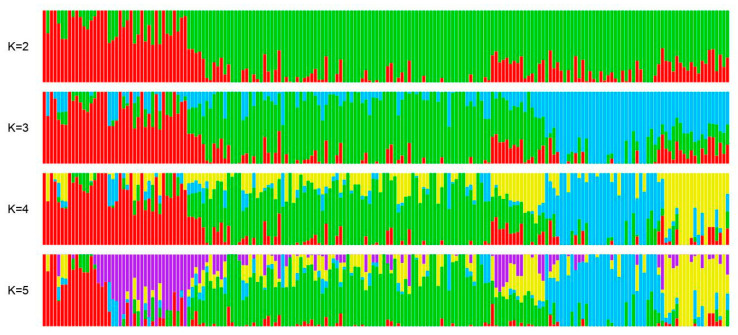
Distribution of the 189 genotypes using SNP markers in groups according to the structure analysis (k = 2 to k = 5). Each individual is represented by a colored bar with a length proportional to the estimated membership to subgroups.

The kinship matrix of the 189 bread wheat lines, excluding the checks, is shown in Figure 2. Lines clustered into two main groups, group 1 consisted of 17, and group 2 of 172 lines. Overall, the lines could be divided into six major subgroups (Figure 2). Over the 100 replicates of ADMIXTURE runs, the CV values had a minimal average at K = 5. Thus, the most probable number of ancestral subpopulations was K = 5 (Figure 3).

#### 2.2.1. Linkage Disequilibrium

Linkage disequilibrium was estimated between the mapped SNPs on each chromosome. The pairwise *r*^2^ values were plotted to the physical distance in the base pair (bp) to infer the extent of LD decay. LD decayed with genetic distance, and it reached 50% of its initial value at just below the 5 Mb. The statistically significant threshold for R^2^ for the whole genome LD was kept at 0.2 (data not shown).

#### 2.2.2. GWAS for PT, PN and CR

Association analysis was carried out using the mixed linear model (MLM). Nineteen different SNPs were significantly associated with CR, PT, and PN resistance (Table 3). Nine SNPs (eight unique) were associated with CR, which were located on chromosomes 1A, 2B, 3A, 4B, 5B, and 7D. Two SNPs were detected for CR1 while four for CR2. Only one marker, S7D_579535886, was associated in the CR_K experiment, S2B_549201894 was associated in the CR_GH, and S2B_708689405 in the CR_Y experiment. Marker ‘S4B_539004405’ was significant in both the CR1 and CR2 experiments. For PT and PN, 12 SNPs (Eleven unique) were detected, seven of which were associated with *P. neglectus* and remaining with *P. thornei* resistance. One marker, ‘S5B_84880275’, showed significant association in the PN1 and PN2 experiment, located on chromosome 5B. Chromosome 5B had the highest number of markers associated with PT1, CR1, CR2, PN1, and PN2. Only one marker (S3A_501892003) was identified for PT2.

## 3. Discussion

Wheat is one of the main crops grown globally as a staple food and source of income for many people. A decrease in wheat yields could result in global food security issues and may lead to hunger in some cases. Wheat production is limited by several abiotic and biotic stresses. Biotic stress factors are a continuous thread to crop production. Although considerable efforts have been undertaken in many breeding programs to increase biotic stress tolerance/resistance, with the newly evolving strains, the race for releasing new cultivars with resistance to biotic stresses should be elevated. Despite the importance of root diseases such as crown rot and root-lesion nematodes, they have received little attention in most breeding programs. Therefore, the knowledge in resistance or tolerance sources against such diseases and molecular understanding is quite limited. In many cases, root diseases are mistakenly recorded as nutrient deficiencies, and proper control measures are not applied.

The development of nematode-resistant wheat was first carried out by Brown and Ellis [24]. Resistant cultivars are the most effective approach to combat root diseases; however, screening and discovering resistance against these diseases is complex. High-throughput and reliable screening methods to speed up the process are needed. In our study, a large panel of CIMMYT wheat lines was evaluated for CR, PN, and PT resistance. The results for growth room, greenhouse, and field evaluations provide a deeper understanding of the type of resistance and correlations between different species and growth conditions. Significant correlations obtained between controlled (GR and GH) and field conditions could speed up the evaluation, screening, and selection processes for root diseases. The three different growth conditions were compared for disease screening, and some levels of correlation were obtained (Table 2). Significant correlations were revealed between CR1 and CR_GH, CR_Y and CR_K, CR1-2, and PN1-2. The resistance of a disease having a significant correlation to another disease could provide indirect selection to that (correlated) resistance/disease. Additionally, heritability was high. Our results suggest moderate-level correlations between different growth conditions. Further studies will be needed to clarify and extend this knowledge to enhance the selection process. Erginbas-Orakci et al. [19] reported similar correlation values for the crown rot disease assessment in a growth room, greenhouse, and field conditions.

A panel of 198 elite spring wheat accessions, including checks, were evaluated against CR, PT, and PN, and significant genetic variation for resistance was obtained. The panel was well balanced, with 31.2% of the lines resistant, 15.3% moderately resistant, 14.8% moderately susceptible, and 38.6% susceptible to CR (Table S2). In total, 30% of the genotypes had similar or higher resistance levels compared to the best check genotype (Croc_1/*Ae. squarrosa* (224)//Opata) in the PT1 experiment, 21% in the PT2, 22% in the PN1, and around 27% PN2 experiment. Similarly, for *Fusarium culmorum,* the GR1 and GR2 experiments had 17%, and 24% of genotypes had lower CR scores compared to the best checks. In the case of the CR_GH experiment, about 11% of genotypes demonstrated a lower CR score, for the field experiment in Yozgat (CR_Y), 18% and Konya (CR_K), 21% of the total germplasm had a lower CR score compared to the best checks.

### Identification of Resistance Sources Using Genome-Wide Association Approach

GWAS is an efficient approach for identifying molecular markers linked to important agronomic traits and disease resistance in bread wheat [4,19,20,23,25,26,27,28]. LD decay is an important factor in GWAS as it gives an idea about the precision of the detected MTAs as well as the number of markers required to cover the genome [29]. More precise MTAs can be detected using populations with fast LD decay. In this study, the LD decayed approximately at 5 Mb, which is much smaller than previously reported in CIMMYT wheat [30,31]. The D genome had fewer markers and thus had comparatively rapid decay compared to A and B genomes, which has also been reported in multiple studies [32,33,34]. These results also show similarity with the results of the wheat association mapping initiative (WAMI) panel [27]. The result of the Chinese winter wheat collection [32,33] has reported genome-specific LD patterns in the wheat genomes reporting that the D genome decayed two to three times slower compared to A and B genomes.

We identified 19 significant MTAs with the root disease resistance on chromosomes 1A, 1B, 2A, 2B, 3A, 4A, 4B, 5B, 5D, and 7D. Of the 19 markers, 8 markers showed association with CR resistance and 11 with PT and PN resistance (Table 3). There were seven different markers associated with the *P. neglectus* resistance, while four markers were associated with PT resistance. There were several reports of crown rot and root-lesion nematode resistance. Collard et al. [35] reported two QTL on chromosome 1A for CR in a doubled haploid (DH) mapping population derived from a cross between cultivars 2-49 (MR) and Janz. Bovill et al. [36] reported QTL linked with CR resistance on chromosome 2B. Here, two QTL were found on chromosome 2B under CR_GH and CR-Y conditions. Wallwork et al. [37] reported QTL for CR resistance (*F. pseudograminearum* and *F. culmorum*) in Kukri (moderately resistant, MR) and Janz (susceptible, S) DH mapping population. They identified a QTL on chromosome 4B, linked to the dwarfing gene *Rht-B1*. However, the dwarfing gene *Rht-B1* is very much fixed in the CIMMYT germplasm. Therefore, the QTL identified in this study on chromosome 4B is most likely not linked to the dwarfing gene. Several reports [38,39] identified CR resistance QTL on chromosomes 3A, 4B, and 5B, which are on the same chromosomes we identified. This similarity is worth further in-depth comparison for the locations of the QTL. The other MTA on chromosome 7D has been reported for the first time, showing association with CR resistance. One marker called S5B_84880275 was significant for PN in both PN1 and PN2 experiments. The seven identified markers being associated with PN resistance were located on five different chromosomes. Of these, the ones on chromosomes 4A and 5B were previously reported by Mulki et al. [20]. In a similar study, Kumar et al. [40] reported significant MTAs, on chromosomes 1B, 1D, 3B, and 6B, explaining 23% to 26% of the total phenotypic variation. The SNP marker S4B_539004405 was obtained in both repeats of CR under growth room conditions. Therefore, chromosomes 4B and 5B look promising since several previous reports and our results highlighted these chromosomes as a resource for CR, PT, and PN resistance alleles.

The most effective way to manage root diseases is to develop resistant wheat germplasm. So, the first step is identifying resistant sources, which can be performed by screening or by taking advantage of marker-assisted selection (if significant large effect QTL are known). In both cases, phenotyping is very important. Results from our study could help to expand our knowledge of the source of resistance in germplasm and genomic regions controlling resistance against crown rot and root-lesion nematodes in wheat. Of the SNPs associated with resistance against CR, PT, and PN, two were consistent across experiments or seasons. The SNP marker S5B_84880275 (R^2^ mean 8.36) was associated with resistance against *P. neglectus* (PN1 and PN2), and S4B_539004405 (R^2^ mean 5.1) was associated with resistance to CR (CR1 and CR2). These two markers were consistently observed across the two different experiments. The favorable allele at S5B_84880275 and S4B_539004405 were found in 36 and 24 lines, respectively. Consistency in these two markers with the acceptable R^2^ values (Table 3) suggests the possibility of use marker-assisted selection (MAS).

Markers associated with certain genomic regions controlling the resistances can improve our understanding of the overall genetic architecture and ability to plan future crosses for the improvement of root traits. Our study showed that 21 SNP markers were significantly associated with the CR, PT, and PN resistance. Genotypes GID6181748, GID6278812, GID6279005, GID6424861, GID6424874, and GID6487731 with S5B_84880275 and S4B_539004405 markers showed moderate to complete resistance against CR, PT and PN. However, there is a need for the validation of these markers before conducting MAS studies. Nevertheless, these genotypes can be considered promising for the transfer of disease-resistance genes into elite material or cultivars. Similar studies need to be carried out on root diseases to enhance the progress of breeding for root diseases and extend the pool of resistance alleles for sustainability in plant protection and environmental safety.

## 4. Materials and Methods

### 4.1. Plant Material and Experimental Procedures

A total of 198 CIMMYT (International Maize and Wheat Improvement Center) spring bread wheat lines, including nine check varieties, were evaluated in this study (Table S1). The lines were selected based on their diverse genetic background and were further purified applying two generations of single-seed descent. The lines were evaluated in multiple experiments. For resistance to root-lesion nematodes (RLN), *Pratylenchus neglectus* (PN) and *P. thornei* (PT), 189 lines and 4 check cultivars were evaluated under growth room (GR) conditions, while for resistance to *F. culmorum* (CR), 189 lines and 5 checks were phenotyped in the GR, greenhouse (GH), and under field conditions. Wheat cultivars “2-49”, “Sunco”, and “Altay” were used as resistant checks, whereas the lines “Seri” and “Suzen” were used as susceptible checks for *F. culmorum*. In addition, the lines “Croc_1/*Ae. squarrosa* (224)//Opata” and “Gs50a” were used as a moderate resistant check for *P. thornei,* whereas the cultivars “Gatcher” and “Suzen” were used as susceptible checks for both *P. thornei* and *P. neglectus* (Table S1) while the line Croc_1/*Ae. squarrosa* (224)//Opata” was used as moderately resistant for both nematodes species.

### 4.2. Growthroom Screening for Root-Lesion Nematodes

Resistance against *P. thornei* and *P. neglectus* were evaluated under GR conditions with two consecutive independent experiments (PN1-2, PT1-2). A single pre-germinated seed was planted in a standard small tube (2.5 cm in diam × 16 cm in length) filled with a sterilized mixture of sand, field soil, and organic matter (70:29:1; *v*/*v*/*v*). The field soil and sand were sieved and sterilized at 110 °C for two h on two consecutive days, whereas the organic matter was sterilized at 70 °C for 5 h. One week after planting, each plant was inoculated with 400 nematodes of either *P. thornei* or *P. neglectus* originating from nematodes reared on carrot discs as described by Dababat et al. [4] and Moody et al. [41]. Trials were performed in 3 replicates and repeated twice. Plants were grown under a day/night photoperiod of 16/8 h at 23 ± 1 °C, and relative humidity of 60%/80% (±5%). The plants were harvested nine weeks after nematode inoculation, shoots were removed, and *P. thornei* and *P. neglectus* individuals were extracted from the roots and soil using the modified Baermann funnel [42]. The total number of *P. thornei* and *P. neglectus* nematodes per plant was calculated based on the number of nematodes in 1 mL suspension multiplied by the total volume and counted under a microscope. Genotypes were divided into five groups based on the number of nematodes per plant, considering the reaction of check varieties used with their known resistant responses to both nematodes species.

### 4.3. Screening for F. culmorum

#### 4.3.1. Inoculum Preparation

The *F. culmorum* isolate was obtained from an infected wheat plant in Kırşehir, Turkey (39°39′709″ N, 34°25′515″ E). A monosporic isolate was transferred to nutrient agar and cultured at 23 ± 1 °C with a 12 h photoperiod for 10 days for spore formation. Bags (35 × 48 cm) filled with wheat bran were moistured and sealed with cotton and then autoclaved at 121 °C for 20 min for three consecutive days. The spore suspension was prepared by adding sterilized distilled water to each Petri dish containing *F. culmorum* culture. The autoclaved bags filled with wheat bran were inoculated with spore suspension under sterilized conditions and were incubated for 2–3 weeks with a 12 h photoperiod and 23 ± 1 °C by shaking until the bran was sufficiently colonized by the fungus [12,19]. The fungus-colonized wheat bran was dried at room temperature and used for GR, GH, and field experiments.

#### 4.3.2. Growth Room Experiments

Resistance against *F. culmorum* was evaluated in the GR with two consecutive experiments (CR1 and CR2). Fungus inoculated wheat bran was suspended with distilled water and filtered using two layers of cheesecloth. Spore concentration was adjusted to 1 × 10^6^ spores mL^−1^, and methylcellulose (0.1%) was added to the spore suspension prior to use. A total of ten seeds per accession were placed on moist blotting papers in sterilized Petri dishes and left to germinate at 22 °C for 2–3 days to obtain similar phenological development. Each seedling was sown in a separate plastic tube (2.5 cm in diam × 16 cm in height) (Ray Leach Cone-tainer^TM^, Stuewe & Sons, Inc., Corvallis, OR, USA) filled with (62 g) potting mix of sterilized sand, soil, and organic manure (50:40:10; *v*/*v*/*v*) and covered with the same substrate. After one week of growth, each seedling (0.5–1 cm above the soil level, including the coleoptile) was inoculated from the base of the stem with *F. culmorum* spore suspension. Tubes inoculated with *F. culmorum* were kept at 23 ± 1 °C for 48 h under high humidity (80–90%) by covering them with plastic sheeting. Following incubation, seedlings were kept in the GR for 42 days (early tillering, until Zadoks growth stage 14 (62)), with a day/night photoperiod of 16/8 h, at 23 ± 1 °C, and relative humidity of 60%/80% (±5%). The plants per each experiment were placed in a randomized complete block design (RCBD) with five replications (1 plant per replicate), and each experiment was repeated twice.

#### 4.3.3. Greenhouse Experiment

To evaluate the resistance in the spring wheat panel against *F. culmorum* in the CR_GH, two seeds of each wheat line were planted in one plastic tube (3.8 cm in diam. × 21 cm in length) (Ray Leach Cone-tainer, Stuewe & Sons, Inc., Corvallis, OR, USA) using the same potting mix as described above, along with 0.5 g fungus-colonized wheat bran (as an inoculum source). Tubes were then put in a stand (RL98; Ray Leach stand, Stuewe & Sons, Inc., Corvallis, OR, USA) placed on the sand to facilitate root growth. Experiments were sufficiently irrigated during the growing season. Plants were exposed to drought stress at maturity to promote disease development. The experiment was set up using RCBD with six replications and two plants per replicate.

#### 4.3.4. Field Experiments

Field experiments were conducted at Yozgat (CR_Y) and Bahri Dağdaş International Agricultural Research Institute in Konya (CR_K), Turkey, under naturally infested field conditions during the 2013–2014 and 2014–2015 growing seasons (October to June). For each line, 5 g of seeds were sown in 1 m rows and infested with an addition of 2 g of fungus-colonized wheat bran. Experiments were arranged in RCBD with three replications. Disease symptoms were scored by randomly picking up 15 individual tillers from each row at the full maturity stage.

### 4.4. Disease Assessment

Seedling resistance (Zadoks growth stage 14) was evaluated from the GR experiments, while for GH and field experiments, plants were evaluated for adult plant resistance. When the diseases were scored, crown rot was scored by one person. While root-lesion nematode scoring was performed by three well-trained people using binoculars. Plants were harvested at the end of each experiment, after 7 weeks in GR, and at full maturity in GH and field experiments, following common practices. Disease scoring was carried out using a modified method of Wildermuth and McNamara [10], following the browning percentage on the crown and the main stem using a numeric scale of 1 to 5 [12,19]. Accordingly, plants were classified as resistant (1: 1–9%), moderately resistant (2: 10–29%), moderately susceptible (3: 30–69%), susceptible (4: 70–89%), and highly susceptible (5: 90–99%) based on % disease ratios.

### 4.5. Data Analyses

Data analyses were performed using QTL Icimapping software [43]. Experiments were conducted under growth room (GR), greenhouse (GH), and field conditions (CR_K and CR_Y) following completely randomized design and randomized complete block design (RCBD) with five replications in the growth room, six replications in the greenhouse, and three replications under field conditions. Analysis of variance (ANOVA) was performed to evaluate the main phenotypic effect of genotype under GR, GH, and field conditions for all disease scores. The broad-sense heritability (H^2^) for all environments was estimated according to Lewien et al. [44]. Descriptive stats for mean, min, and max values were evaluated using JASP software (JASP v. 0.16, JASP Team 2021). Correlation coefficients between GR, GH, and field experiments for CR and RLN were estimated with Pearson’s correlation analysis using JASP software.

### 4.6. Genome-Wide Association Mapping

#### 4.6.1. DNA Extraction and Genotyping

Genomic DNA was extracted from bulked leaves of 10 two-week-old seedlings leaves using a cetyltrimethylammonium bromide procedure [45] modified according to CIMMYT protocols [46]. Lines were genotyped using genotyping-by-sequencing as described in Poland et al. [47]. After cleaning the raw data sets by removing the markers having more than 20% missing data and the minor allele frequency (MAF) < 5%, 4056 SNP markers were used for further analyses.

#### 4.6.2. Population Structure Analysis

Population structure was assessed using ADMIXTURE software with 10 cross-validations and k ranging between 2 and 20 [48]. Overall, 100 repeats were conducted, and the cross-validation values were averaged across these repeats for each k. The most probable k value was defined as the k that had the lowest cross-validation average value. As ADMIXTURE analysis assumes SNPs to be unlinked or under linkage equilibrium, SNPs were pruned at an R^2^ value equal to 0.5 using PLINK [49].

#### 4.6.3. Linkage Disequilibrium and GWAS

Linkage disequilibrium (LD) was calculated using polymorphic markers with allele frequency higher than 5% using a custom R script [50]. LD was assessed as squared allele frequency correlation (*r*^2^). GWAS was conducted using the mixed linear model (MLM) implemented in the Genome Association and Prediction Integrated Tool (GAPIT) in R [51]. The genomic relationship matrix was fitted as a random covariate, while the principal component (PC) analysis was fitted as fixed covariates in the MLM analysis. The optimal numbers of PCs were determined using the Bayesian information criterion implemented in GAPIT. A significant threshold (0.000015) was defined using the method described in Tang et al. [51]. This method estimates the effective number of markers or the independent number of markers, so it can consider the dependency of SNPs due to LD.

## Figures and Tables

**Table 1:** Descriptive statistics, variance analysis (ANOVA), and broad-sense heritability (H^2^) values for growth room (PT1-2 and PN1-2 and CR1-2), greenhouse (CR), and field (CR_Y and CR_K) in Yozgat and Konya experiments, respectively.

**(a) *Pratylenchus* Species**	**Mean** ** ± SD**	**CV**	**Min.**	**Max.**	***p*-Value**	**H^2^**
PT1	506.550 ± 25.004	0.679	25	1925	***	0.9034
PT2	849.746 ± 43.132	0.698	92	3104	***	0.9307
PN1	789.947 ± 47.195	0.821	25	3149	***	0.9734
PN2	948.048 ± 55.904	0.811	75	3653	***	0.9874
**(b) Crown Rot** **(*Fusarium culmorum*)**	**Mean** ** ± SD**	**CV**	**Min.**	**Max.**	***p*-Value**	**H^2^**
CR1	2.929 ± 0.050	0.233	1.20	4.40	***	0.9125
CR2	2.939 ± 0.055	0.257	1.00	4.60	***	0.9276
CR_GH	3.182 ± 0.047	0.204	1.00	4.33	***	0.8285
CR_Y	2.920 ± 0.042	0.199	1.67	4.00	***	0.7952
CR_K	3.033 ± 0.051	0.231	1.00	4.33	***	0.8883

***: significant at *p* < 0.001 level. *Pratylenchus thornei* (PT) and *Pratylenchus neglectus* (PN), *Fusarium culmorum* (CR), SD: standard deviation, CV: coefficient of variation.

**Table 2:** Overall means from growth room (PN1-2, PT1-2, and CR1-2), greenhouse (CR_GH), and field (CR_Y and CR_K) experiments were used separately for Pearson correlation analysis.

	PT1	PT2	PN1	PN2	CR1	CR2	CR_GH	CR_Y
PT2	0.290 ***							
PN1	0.309 ***	0.341 ***						
PN2	0.219 **	0.323 ***	0.903 ***					
CR1	0.073	0.061	0.159 *	0.164 *				
CR2	0.116	0.044	0.254 ***	0.227 **	0.724 ***			
CR_GH	0.078	−0.083	0.028	0.02	0.188 **	0.054		
CR_Y	0.044	−0.138	−0.047	0.02	0.11	0.089	0.108	
CR_K	−0.098	−0.213 **	0.260 ***	−0.232 **	0.023	−0.07	0.13	0.532 ***

* significant at *p* < 0.05, ** significant at *p* < 0.01, *** significant at *p* < 0.001.

**Table 3:** Significant markers associated with resistance to *Fusarium culmorum*, *Pratylenchus neglectus*, and *Pratylenchus thornei* according to genome-wide association (GWAS) analysis of 189 spring wheat accession under growth room (PN1-2, PT1-2, and CR1-2), greenhouse (GH), and field conditions in both Yozgat and Konya (CR_Y and CR_K), respectively.

	Trait	SNP #	*p*-Value	MAF	R^2^	Effect
*F. culmorum*	CR_GH	S2B_549201894	9.8 × 10^−6^	0.11	11.08	0.40
	CR_K	S7D_579535886	2.1 × 10^−4^	0.05	7.2	0.51
	CR_Y	S2B_708689405	4.0 × 10^−4^	0.14	6.87	0.24
	CR1	S3A_738043010	1.9 × 10^−4^	0.06	7.54	0.44
	CR1	S4B_539004405	2.0 × 10^−4^	0.12	7.50	0.34
	CR2	S1A_10214692	3.2 × 10^−4^	0.17	7.00	0.27
	CR2	S1A_38715697	5.0 × 10^−5^	0.26	8.99	0.27
	CR2	S4B_539004405	4.0 × 10^−5^	0.12	9.22	0.39
	CR2	S5B_598755542	2.1 × 10^−4^	0.07	7.42	−0.41
*P. neglectus*	PN1	S2A_24002740	7.7 × 10^−5^	0.17	6.3	312.9
	PN1	S5B_84880275	4.3 × 10^−4^	0.20	5.0	231.2
	PN2	S1A_41054531	3.6 × 10^−4^	0.15	5.6	271.5
	PN2	S2A_154414007	3.8 × 10^−4^	0.29	5.6	−290.3
	PN2	S2A_160931482	2.9 × 10^−4^	0.23	5.8	274.6
	PN2	S4A_48532477	4.0 × 10^−4^	0.18	5.5	256.8
	PN2	S5B_84880275	4.2 × 10^−4^	0.20	5.2	269.4
	PN2	S5D_541692475	2.4 × 10^−4^	0.07	6.0	−388.2
*P. thornei*	PT1	S1B_366267523	7.3 × 10^−5^	0.05	8.4	−233.2
	PT1	S3A_732049884	2.2 × 10^−4^	0.17	7.2	124.9
	PT1	S5B_38505289	4.3 × 10^−4^	0.12	6.6	−143.0
	PT2	S3A_501892003	4.5 × 10^−4^	0.07	5.7	307.4

**#:** the SNP name includes the chromosome and its position, MAF: minor allele frequency, R^2^: coefficient of determination.

## Data Availability

All data and Supplementary Data are included with this submission.

## Supplementary Materials

The following supporting information can be downloaded at: https://www.mdpi.com/article/10.3390/life12030372/s1, Table S1. (a) 189 bread wheat genotypes and controls genotypes used in this study, (b) Check spring wheat lines used in the study; Table S2. Arithmetic means of 189 spring wheat lines assessed under growth room conditions, greenhouse, and field conditions for root-lesion nematodes (PT and PN), and crown rot (CR); Table S3. Arithmetic means of check spring wheat lines assessed under growth room (GR) conditions, greenhouse (GH), and field conditions (CR_Y and CR_K) for root-lesion nematodes (PT and PN) and/or crown rot (CR).
