# Supplementary material for: Genome-Wide Association Study of Root-Lesion Nematodes Pratylenchus Species and Crown Rot Fusarium culmorum in Bread Wheat
Source: Life (Basel). 2022 Mar 4;12(3):372. doi: 10.3390/life12030372 (PMC8949321; doi:10.3390/life12030372)
Supplement: Supplementary file 1 [file life-12-00372-s001.zip › life-1568265-supplementary.pdf]

life

MDPI

Article

Genome-Wide Association Study of Root-Lesion Nematodes Pratylenchus Species and Crown Rot *Fusarium culmorum* in Bread Wheat

Quahir Sohail [1], Gul Erginbas-Orakcı [2], Fatih Ozdemir [3], Abdulqader Jighly [4], Susanne Dreisigacker [5], Harun Bektas [6], Nevzat Birişik [7], Hakan Ozkan [8,*] and Abdelfattah A. Dababat [2,*]

Supplementary Materials:

Table S1. (a) 189 bread wheat genotypes and controls genotypes used in this study, (b) Check spring wheat lines used in the study.

		(a) 189 bread wheat genotypes and controls genotypes used in this study
**S.#**	**GID**	**Pedigree**
1	6424869	VORB*2/3/PFAU/WEAVER//KIRITATI
2	6487396	CNDO/R143//ENTE/MEXI_2/3/AEGILOPS SQUARROSA(TAUS)/4/WEAVER/5/2*JANZ/6/SKAUZ/BAV92
3	6174901	ALTAR 84/AE.SQUARROSA (221)//3*BORL95/3/URES/JUN//KAUZ/4/WBLL1/5/MILAN/S87230//BAV92
4	6424422	KA/NAC//TRCH/4/MILAN/KAUZ//DHARWAR DRY/3/BAV92
5	6681793	ND643/2*WBLL1/4/WHEAR/KUKUNA/3/C80.1/3*BATAVIA//2*WBLL1
6	6624540	C80.1/3*BATAVIA//2*WBLL1/3/EMB16/CBRD//CBRD/4/MILAN/KAUZ//DHARWAR DRY/3/BAV92
7	6624544	C80.1/3*BATAVIA//2*WBLL1/3/EMB16/CBRD//CBRD/4/CHEWINK #1
8	6279929	SOISSONS/KUKUNA//WBLL1*2/TUKURU
9	6176225	FRET2/TUKURU//FRET2/3/MUNIA/CHTO//AMSEL/4/FRET2/TUKURU//FRET2
10	6280037	HUANIL/5/2*CNO79//PF70354/MUS/3/PASTOR/4/BAV92
11	6179276	TACUPETO F2001/6/CNDO/R143//ENTE/MEXI_2/3/AEGILOPS SQUARROSA (TAUS)/4/WEAVER/5/PASTOR/7/ROLF07
12	6176334	ROLF07*2/5/REH/HARE//2*BCN/3/CROC_1/AE.SQUARROSA (213) //PGO/4/HUITES
13	6176213	BAV92//IRENA/KAUZ/3/HUITES/4/2*ROLF07
14	6175962	ATTILA*2/PBW65*2//KACHU
15	6280483	BWD-4/3/ATTILA/BAV92//PASTOR/4/ATTILA*2/PBW65
16	6178029	PFAU/WEAVER//KIRITATI/3/FRET2/TUKURU//FRET2/4/FRET2/TUKURU//FRET2
17	6178997	QUAIU/5/FRET2*2/4/SNI/TRAP#1/3/KAUZ*2/TRAP//KAUZ
18	6175213	ATTILA*2/PBW65*2/5/REH/HARE//2*BCN/3/CROC_1/AE.SQUARROSA (213) //PGO/4/HUITES
19	6175211	ATTILA*2/PBW65*2/5/REH/HARE//2*BCN/3/CROC_1/AE.SQUARROSA (213)//PGO/4/HUITES
20	6181746	WBLL1*2/BRAMBLING//JUCHI
21	6174889	BECARD/KACHU
22	6179291	WBLL1*2/KUKUNA//KIRITATI/3/WBLL1*2/KUKUNA
23	6568075	KACHU//KIRITATI/2*TRCH
24	6566562	CROC_1/AE.SQUARROSA (205) //BORL95/3/PRL/SARA//TSI/VEE#5/4/FRET2/5/CHONTE/6/INQALAB 91*2/KUKUNA//KIRITATI
25	6463812	BECARD #1/5/KIRITATI/4/2*SERI.1B*2/3/KAUZ*2/BOW//KAUZ

26	6682939	WBLL1*2/BRAMBLING*2//BAVIS
27	6179475	PRL/2*PASTOR*2//VORB
28	6280233	BABAX/LR39//BABAX/3/VORB/4/SUNCO/2*PASTOR
29	6280250	KRICHAUFF/2*PASTOR//SOKOLL
30	6279600	KANZ/5/CNO79//PF70354/MUS/3/PASTOR/4/BAV92/6/PRL/SARA//TSI/VEE#5
31	6424850	D67.2/PARANA 66.270//AE.SQUARROSA (320)/3/CUNNINGHAM/4/SKAUZ/BAV92
32	6280455	KRICHAUFF/2*PASTOR/4/MILAN/KAUZ//PRINIA/3/BAV92
33	6624427	KA/NAC//TRCH/3/VORB
34	6624426	KA/NAC//TRCH/3/VORB
35	6424871	VORB*2/3/PFAU/WEAVER//KIRITATI
36	6174847	TACUPETO F2001*2/BRAMBLING//WBLL1*2/BRAMBLING
37	6178935	WBLL1*2/TUKURU//FN/2*PASTOR/3/FRET2/KIRITATI
38	6280219	C80.1/3*BATAVIA//2*WBLL1/4/D67.2/PARANA 66.270//AE.SQUARROSA (320) /3/CUNNINGHAM/5/T.DICOCCON PI225332/AE.SQUARROSA (895) //WBLL1/3/2*WBLL1
39	6176013	ROLF07/4/BOW/NKT//CBRD/3/CBRD/5/FRET2/TUKURU//FRET2
40	6175947	WBLL1*2/KURUKU//HEILO
41	6683482	KIRITATI//HUW234+LR34/PRINIA/3/BAJ #1
42	6683480	KIRITATI//HUW234+LR34/PRINIA/3/BAJ #1
43	6569050	DANPHE #1*2/CHYAK
44	6684188	MUTUS*2//ND643/2*WBLL1
45	6684341	Unknown
46	6179370	KSW/5/2*ALTAR 84/AE.SQUARROSA (221)//3*BORL95/3/URES/JUN//KAUZ/4/WBLL1
47	6174903	ALTAR 84/AE.SQUARROSA (221)//3*BORL95/3/URES/JUN//KAUZ/4/WBLL1/5/MILAN/S87230//BAV92
48	6177552	BAJ #1/AKURI
49	6174877	WBLL1*2/4/SNI/TRAP#1/3/KAUZ*2/TRAP//KAUZ/5/BAJ #1
50	6178973	PFAU/SERI.1B//AMAD/3/WAXWING/4/BABAX/LR42//BABAX*2/3/KURUKU
51	6174860	FRANCOLIN #1//WBLL1*2/BRAMBLING
52	6175411	WAXWING*2/HEILO
53	6176409	ATTILA*2/PBW65*2//W485/HD29
54	6174858	FRANCOLIN #1/WBLL1
55	6682916	FRNCLN/3/KIRITATI//HUW234+LR34/PRINIA/4/FRANCOLIN #1
56	6681419	WAXWING*2/TUKURU//2*FRNCLN
57	6682884	FRNCLN/NIINI #1//FRANCOLIN #1
58	6681477	FRANCOLIN #1/CHONTE//FRNCLN
59	6682434	WAXWING*2/TUKURU/3/2*WHEAR/VIVITSI//WHEAR
60	6417653	PFAU/SERI.1B//AMAD/3/WAXWING/4/BECARD
61	6175963	ATTILA*2/PBW65*2//KACHU
62	6280099	PBW343*2/KUKUNA//WBLL1*2/KUKUNA
63	6280103	PBW343*2/KUKUNA//WBLL1*2/KUKUNA
64	6280120	SLVS/ATTILA//WBLL1/4/FRAME*2/3/URES/JUN//KAUZ
65	6280121	SLVS/ATTILA//WBLL1/4/FRAME*2/3/URES/JUN//KAUZ
66	6279825	SLVS/ATTILA//WBLL1*2/3/GONDO/CBRD
67	6279829	SLVS/ATTILA//WBLL1*2/3/GONDO/CBRD
68	6415172	BECARD/FRNCLN
69	6424312	TOB/ERA//TOB/CNO67/3/PLO/4/VEE#5/5/KAUZ/6/FRET2/7/PASTOR//MILAN/KAUZ/3/BAV92
70	6424301	TOB/ERA//TOB/CNO67/3/PLO/4/VEE#5/5/KAUZ/6/FRET2/7/PASTOR//MILAN/KAUZ/3/BAV92
71	6424434	KA/NAC//TRCH/3/DANPHE #1

72	6682171	QUAIU*2/KINDE
73	6624392	METSO/ER2000/5/2*SERI*3//RL6010/4*YR/3/PASTOR/4/BAV92
74	6487493	MON/IMU//ALD/PVN/3/BORL95/4/OASIS/2*BORL95/5/SKAUZ/BAV92
75	6487536	TOB/ERA//TOB/CNO67/3/PLO/4/VEE#5/5/KAUZ/6/FRET2/7/MINO
76	6177412	WBLL1*2/KKTS//KINGBIRD #1
77	6177411	WBLL1*2/KKTS//KINGBIRD #1
78	6327724	BABAX/LR42//BABAX*2/3/KURUKU/4/KINGBIRD #1
79	6624455	METSO/ER2000//MONARCA F2007/3/WBLL1*2/KKTS
80	6424619	METSO/ER2000/3/EMB16/CBRD//CBRD
81	6177544	MILAN/S87230//BAV92/3/AKURI
82	6341870	MILAN/S87230//BAV92*2/3/AKURI
83	6356457	REH/HARE//2*BCN/3/CROC_1/AE.SQUARROSA (213) //PGO/4/HUITES/5/T.DICOCCON PI94624/AE.SQUARROSA(409)//BCN/6/REH/HARE//2*BCN/3/CROC_1/AE.SQUARROSA (213)//PGO/4/HUITES/7/ MILAN/S87230//BAV92
84	6177447	KBIRD//WBLL1*2/KURUKU
85	6179542	WBLL1*2/KURUKU/4/BABAX/LR42//BABAX*2/3/KURUKU
86	6175234	WBLL1*2/KURUKU/6/CNDO/R143//ENTE/MEXI_2/3/AEGILOPS SQUARROSA (TAUS)/4/WEAVER/5/2*JANZ/7/WBLL1*2/KURUKU
87	6179538	WBLL1*2/4/YACO/PBW65/3/KAUZ*2/TRAP//KAUZ*2/5/CHUANMAI 32
88	6179255	WBLL1*2/4/BABAX/LR42//BABAX/3/BABAX/LR42//BABAX
89	6174867	WBLL1*2/BRAMBLING/5/WBLL1*2/4/SNI/TRAP#1/3/KAUZ*2/TRAP//KAUZ
90	6179457	TACUPETO F2001//WBLL1*2/KKTS/3/WBLL1*2/BRAMBLING
91	6179465	WBLL1*2/TUKURU*2//KRONSTAD F2004
92	6179463	WBLL1*2/KURUKU//KRONSTAD F2004/3/WBLL1*2/BRAMBLING
93	6334696	WBLL4/KUKUNA//WBLL1/3/WBLL1*2/BRAMBLING
94	6336692	PANDORA//WBLL1*2/BRAMBLING/3/WBLL1*2/BRAMBLING
95	6335276	MILAN/S87230//BAV92/3/WBLL1*2/BRAMBLING/4/WBLL1*2/BRAMBLING
96	6418562	TUKURU//BAV92/RAYON/3/WBLL1*2/BRAMBLING/4/WBLL1*2/BRAMBLING
97	6624543	C80.1/3*BATAVIA//2*WBLL1/3/EMB16/CBRD//CBRD/4/MILAN/KAUZ//DHARWAR DRY/3/BAV92
98	6624541	C80.1/3*BATAVIA//2*WBLL1/3/EMB16/CBRD//CBRD/4/MILAN/KAUZ//DHARWAR DRY/3/BAV92
99	6341533	PFAU/WEAVER*2//TUKURU/4/BABAX/LR42//BABAX*2/3/KURUKU/5/QUAIU
100	6181747	BABAX/LR42//BABAX/3/BABAX/LR42//BABAX/4/ATTILA/2*PASTOR/5/QUAIU #3
101	6181748	BABAX/LR42//BABAX/3/BABAX/LR42//BABAX/4/ATTILA/2*PASTOR/5/QUAIU #3
102	304660	LOCAL CHECK
103	6418598	BABAX/LR42//BABAX*2/3/KUKUNA/4/CROSBILL #1/5/BECARD
104	6418597	BABAX/LR42//BABAX*2/3/KUKUNA/4/CROSBILL #1/5/BECARD
105	6001673	BOW/VEE/5/ND/VG9144//KAL/BB/3/YACO/4/CHIL/6/CASKOR/3/CROC_1/AE.SQUARR OSA (224)//OPATA/7/PASTOR//MILAN/KAUZ/3/BAV92
106	6336494	KIRITATI/WBLL1/4/2*BABAX/LR42//BABAX*2/3/KURUKU
107	6336482	KIRITATI/WBLL1//MESIA/3/KIRITATI/WBLL1
108	6342011	BAJ #1*2/WHEAR
109	6415095	VILLA JUAREZ F2009/CHYAK
110	6417794	ITP40/AKURI
111	6416942	TACUPETO F2001*2/KIRITATI//VILLA JUAREZ F2009
112	6417113	BECARD/QUAIU #1
113	6417117	BECARD/QUAIU #1
114	6417213	BECARD//ND643/2*WBLL1

115	6418480	WBLL1*2/BRAMBLING//KINGBIRD #1
116	6415761	FRET2*2/BRAMBLING//BECARD/3/WBLL1*2/BRAMBLING
117	6683296	PRL/2*PASTOR/3/PFAU/WEAVER*2//CHAPIO
118	6681297	PRL/2*PASTOR/4/CHOIX/STAR/3/HE1/3*CNO79//2*SERI/5/KIRITATI/2*TRCH/6/PRL/2*PASTOR/4/ CHOIX/STAR/3/HE1/3*CNO79//2*SERI
119	6624336	SUNCO.6/FRAME//PASTOR/3/PAURAQ
120	6418261	BECARD/3/PASTOR//MUNIA/ALTAR 84
121	6177947	UP2338*2/VIVITSI/3/FRET2/TUKURU//FRET2/4/MISR 1
122	6178556	PRL/2*PASTOR*2//FH6-1-7
123	6280591	SOKOLL//INQALAB 91*2/KUKUNA
124	6280415	T.TAU.83.2.29/ATTILA//ATTILA/3/EXCALIBUR
125	6279632	BABAX/KS93U76//BABAX/3/2*SOKOLL
126	6279631	BABAX/KS93U76//BABAX/3/2*SOKOLL
127	6424403	KA/NAC//TRCH/3/VORB
128	6424400	KA/NAC//TRCH/3/VORB
129	6624413	KA/NAC//TRCH/3/VORB
130	6424865	VORB*2/3/PFAU/WEAVER//KIRITATI
131	6176360	WBLL1/FRET2//PASTOR*2/3/MURGA
132	6343460	PFAU/MILAN//FISCAL/3/VORB/4/MILAN/S87230//BAV92
133	5435924	W15.92/4/PASTOR//HXL7573/2*BAU/3/WBLL1
134	6278943	VORB/SOKOLL
135	6177174	PFAU/SERI.1B//AMAD/3/WAXWING/4/HUIRIVIS #1
136	6177148	TRCH/HUIRIVIS #1
137	6177648	PBW343*2/KUKUNA//TECUE #1
138	6179222	PBW343*2/KUKUNA*2//FRTL/PIFED
139	6179227	PBW343*2/KUKUNA*2//FRTL/PIFED
140	6624440	BERKUT/MUU//DANPHE #1
141	6624418	KA/NAC//TRCH/3/DANPHE #1
142	6175662	SITE/MO//PASTOR/3/TILHI/4/WAXWING/KIRITATI
143	6624365	1447/PASTOR//KRICHAUFF/3/PAURAQ
144	1987914	HIDDAB
145	72533	PROINTA FEDERAL
146	109278	DHARWAR DRY
147	6178533	SAUAL/YANAC//SAUAL
148	6176829	SAUAL/3/ACHTAR*3//KANZ/KS85-8-4/4/SAUAL
149	6175076	NAC/TH.AC//3*PVN/3/MIRLO/BUC/4/2*PASTOR/5/KACHU/6/KACHU
150	6175067	NAC/TH.AC//3*PVN/3/MIRLO/BUC/4/2*PASTOR/5/KACHU/6/KACHU
151	6424629	EMB16/CBRD//CBRD/4/BETTY/3/CHEN/AE.SQ//2*OPATA
152	6175707	MURGA/KRONSTAD F2004
153	6176368	KACHU #1/4/CROC_1/AE.SQUARROSA(205)//BORL95/3/2*MILAN/5/KACHU
154	6417583	KACHU/KIRITATI
155	6416954	KACHU/KINDE
156	6177598	KINGBIRD #1//INQALAB 91*2/TUKURU
157	6174884	BECARD/KACHU
158	6174886	BECARD/KACHU
159	6415858	KACHU/BECARD//WBLL1*2/BRAMBLING
160	6337034	FRNCLN*2/TECUE #1
161	6337327	PFAU/SERI.1B//AMAD/3/WAXWING/4/AKURI/5/PFAU/SERI.1B//AMAD/3/WAXWING
162	6176914	MUNAL #1/FRANCOLIN #1
163	6176308	ATTILA*2/PBW65*2//MURGA

164	6176558	ATTILA*2/PBW65*2/4/BOW/NKT//CBRD/3/CBRD
165	6179562	PBW343*2/KHVAKI*2//YANAC
166	6624429	QING HAIBEI/WBLL1//BRBT2/3/PAURAQ
167	6332960	KIRITATI/WBLL1//FRANCOLIN #1
168	6174892	FRANCOLIN #1//WBLL1*2/KURUKU
169	6179347	FRANCOLIN #1/4/BABAX/LR42//BABAX*2/3/KURUKU
170	6415924	PFAU/SERI.1B//AMAD/3/WAXWING*2/4/TECUE #1
171	6416482	ND643/2*TRCH/3/MILAN/S87230//BAV92/4/PFAU/SERI.1B//AMAD/3/WAXWING
172	6414818	PBW343*2/KUKUNA/3/PASTOR//CHIL/PRL/4/GRACK
173	2430154	PBW343
174	6278982	DUCULA/GONDO//SOKOLL
175	6278849	H45/4/KRICHAUFF/FINSI/3/URES/PRL//BAV92
176	6279585	INQALAB 91*2/KUKUNA/4/TC14/2*HTG//DUCULA/3/PRINIA
177	6424861	KRICHAUFF/2*PASTOR//2*SOKOLL
178	6278811	D67.2/PARANA 66.270//AE.SQUARROSA (320)/3/CUNNINGHAM/4/VORB
179	6278812	D67.2/PARANA 66.270//AE.SQUARROSA (320)/3/CUNNINGHAM/4/VORB
180	3822784	PRL/2*PASTOR
181	6280583	CHIH95.7.4//INQALAB 91*2/KUKUNA
182	6280588	SOKOLL//INQALAB 91*2/KUKUNA
183	6280393	VORB/6/CPI8/GEDIZ/3/GOO//ALB/CRA/4/AE.SQUARROSA (208)/5/2*WESTONIA/7/CPI8/GEDIZ/3/GOO//ALB/CRA/4/AE.SQUARROSA (208)/5/2*WESTONIA
184	6280381	VORB/4/D67.2/PARANA 66.270//AE.SQUARROSA (320)/3/CUNNINGHAM/5/D67.2/PARANA 66.270//AE.SQUARROSA (320)/3/CUNNINGHAM
185	6487731	VORB*2/5/CROC_1/AE.SQUARROSA(224)//OPATA/3/RAC655/4/SLVS/PASTOR
186	3855011	VOROBEY
187	6424874	VORB*2/3/PFAU/WEAVER//KIRITATI
188	6278810	D67.2/PARANA 66.270//AE.SQUARROSA (320)/3/CUNNINGHAM/4/VORB
189	6279005	VORB/3/T.DICOCCON PI94625/AE.SQUARROSA (372)//3*PASTOR

(b) Check spring wheat lines used in the study

S.#	GID	Pedigree
190	-	2 49 (Check-Cr,Mr)
191	-	Sunco (Check-Cr,Ms)
192	-	Altay(Check-Cr,Mr)
193	-	Seri(Check-Cr,S)
194	-	Suzen(Check-Cr,S)
195	-	CROC_1/*Ae. squarrosa* (224)//OPATA -(Check-Pt,Pn-Mr)
196	-	Gatcher -(Check-Pt,Pn-S)
197	-	Gs50a-(Check-Pt-Mr, Pn-S)
198	-	Seri -(Check-Pt,Pn-S)

Table S2. Arithmetic means of 189 spring wheat lines assessed under growth room conditions, greenhouse, and field conditions for root-lesion nematodes (PT and PN), and crown-rot (CR).

		Pratylenchus thornei		*Pratylenchus neglectus*		*Fusarium culmorum*				
		Growth room (GR)				*Growth room (GR)*		*Greenhouse (GH)*	*Field*	
S.#	GID	PT-1	PT-2	PN-1	PN-2	CR-1	CR-2	CR_GH	CR_Y	CR_K
1	6424869	628	755	635	525	3.00	3.00	3.33	2.00	2.00
2	6487396	146	131	317	907	3.40	3.20	3.00	3.00	3.00
3	6174901	423	1402	1680	1220	4.20	4.60	1.00	2.00	2.00

4	6424422	760	844	1677	2513	3.00	4.40	3.67	3.33	4.00
5	6681793	1309	1382	1100	1287	3.40	3.60	3.67	2.00	2.00
6	6624540	503	1720	823	933	2.00	2.40	3.67	1.67	2.00
7	6624544	389	1702	1537	2007	2.00	2.40	3.00	4.00	3.00
8	6279929	216	248	256	444	3.20	3.00	3.33	2.33	2.33
9	6176225	471	1291	666	860	3.00	4.00	3.67	2.00	2.00
10	6280037	236	1750	280	400	3.60	3.00	3.33	3.00	3.33
11	6179276	1356	580	473	435	3.40	3.60	4.00	3.33	3.33
12	6176334	62	92	151	140	3.60	3.40	3.00	2.67	4.00
13	6176213	671	836	2317	2400	3.20	3.20	3.00	3.33	3.00
14	6175962	102	452	42	736	4.00	2.00	3.33	3.33	3.67
15	6280483	270	632	150	865	3.00	3.20	3.00	3.67	3.33
16	6178029	1260	830	680	530	2.60	2.60	3.00	3.33	3.33
17	6178997	57	164	30	180	2.40	3.40	2.33	2.67	4.00
18	6175213	416	400	210	250	1.20	1.80	1.67	3.00	3.00
19	6175211	245	180	304	305	1.40	1.80	3.00	3.00	3.00
20	6181746	208	120	280	224	2.60	2.40	3.67	3.00	3.33
21	6174889	190	170	129	120	3.50	3.40	3.33	3.67	4.00
22	6179291	972	550	320	307	3.80	3.00	3.67	3.33	3.00
23	6568075	711	1109	1203	1353	3.20	3.00	4.00	2.00	1.67
24	6566562	369	1687	354	747	3.60	4.00	4.33	2.00	2.00
25	6463812	409	1207	400	707	2.20	2.40	1.67	3.33	3.33
26	6682939	434	684	1109	1307	3.40	3.20	3.67	3.00	3.33
27	6179475	768	592	625	400	2.20	2.20	3.33	3.00	3.33
28	6280233	112	160	244	345	2.40	2.60	3.33	2.33	2.00
29	6280250	736	715	790	908	1.60	1.40	3.33	3.33	2.67
30	6279600	315	1028	520	755	3.40	3.20	3.33	3.67	3.33
31	6424850	784	1204	469	608	3.00	3.00	3.33	3.67	3.33
32	6280455	572	2620	336	496	3.40	3.00	3.33	2.67	2.33
33	6624427	363	573	857	1860	3.00	3.00	2.00	3.00	2.00
34	6624426	214	1351	1057	1687	3.60	4.40	3.00	3.00	2.00
35	6424871	676	910	104	153	3.40	3.00	3.00	2.33	2.33
36	6174847	65	130	307	535	3.40	3.60	2.33	3.00	3.67
37	6178935	215	916	319	220	2.00	2.40	3.33	2.33	2.33
38	6280219	150	248	108	135	3.40	3.40	3.67	3.00	3.67
39	6176013	557	1287	1317	1440	4.20	4.40	3.33	4.00	3.33
40	6175947	89	222	65	470	2.00	2.00	3.00	3.00	4.00
41	6683482	720	1402	1163	1040	1.40	1.20	2.00	3.00	3.33
42	6683480	509	1247	171	507	2.40	2.40	2.00	3.67	2.00
43	6569050	426	2529	1171	1387	2.60	2.60	1.67	2.00	2.00
44	6684188	586	1667	1246	1473	1.40	1.60	3.00	2.00	2.00
45	6179370	928	1060	632	464	3.40	3.40	2.00	3.67	3.00
46	6174903	175	138	498	572	2.00	2.20	3.00	3.00	3.33
47	6177552	903	1260	1020	1260	3.60	3.40	3.67	3.00	3.00
48	6174877	900	944	1017	1033	3.20	3.40	4.33	2.67	2.00
49	6178973	162	131	219	535	3.40	3.40	3.00	3.00	3.33
50	6174860	1197	262	914	1093	3.80	4.00	4.00	3.00	2.00
51	6175411	206	514	448	472	3.00	3.20	3.67	3.00	3.67
52	6176409	40	839	25	145	3.40	3.40	3.33	3.00	3.67
53	6174858	41	146	325	412	3.20	3.40	2.33	3.00	2.67
54	6682916	629	1002	951	1267	2.00	2.40	4.00	2.00	2.00

55	6681419	540	1804	1131	1073	3.00	2.40	3.33	2.00	3.00
56	6682884	437	1344	1254	1607	3.00	3.00	3.33	1.67	2.00
57	6681477	866	2650	1577	1707	2.60	3.00	3.67	2.00	2.00
58	6682434	291	3060	1126	1153	3.00	2.40	3.00	2.00	3.33
59	6417653	891	480	3149	3653	4.40	3.20	2.33	3.00	3.33
60	6175963	529	844	1006	2173	4.00	3.60	3.67	4.00	3.67
61	6280099	304	840	584	630	3.20	3.80	3.33	3.67	3.00
62	6280103	204	1430	205	284	2.40	1.80	3.67	3.00	4.00
63	6280120	790	1775	700	292	1.80	1.80	2.67	3.33	3.67
64	6280121	284	627	432	584	3.40	3.20	3.50	2.33	2.00
65	6279825	220	1040	402	635	2.60	2.60	3.67	4.00	3.67
66	6279829	435	1155	545	370	3.60	3.40	3.33	3.67	3.33
67	6415172	751	1420	1049	1453	3.80	4.20	3.00	3.33	3.67
68	6424312	740	704	834	1020	3.20	3.60	2.00	3.33	3.00
69	6424301	683	1722	1391	1307	3.20	3.60	2.33	4.00	4.00
70	6424434	597	711	731	740	2.40	2.40	3.00	2.00	2.00
71	6682171	731	2191	234	633	2.00	2.40	2.00	2.00	3.33
72	6624392	771	940	1774	1453	3.40	3.40	3.00	3.00	3.00
73	6487493	569	564	1306	1473	2.60	2.40	3.67	3.00	3.00
74	6487536	397	871	1791	1913	3.60	3.60	4.00	3.67	3.67
75	6177412	720	935	348	475	3.40	3.00	3.33	3.33	4.00
76	6177411	614	488	232	105	2.40	2.20	3.33	2.00	3.67
77	6327724	649	1153	1891	2353	3.40	3.00	4.00	3.67	3.00
78	6624455	217	527	2191	2847	3.00	3.00	3.00	3.33	3.00
79	6424619	363	643	797	1033	3.20	3.40	3.67	3.00	1.67
80	6177544	663	987	2203	2333	2.60	3.00	3.67	3.00	3.00
81	6341870	311	822	411	460	2.40	1.00	4.33	2.00	3.33
82	6356457	714	360	486	480	2.60	3.60	2.00	3.00	2.00
83	6177447	358	790	501	350	3.60	2.60	3.00	3.00	3.67
84	6179542	200	175	440	576	1.60	1.80	3.00	2.67	2.33
85	6175234	570	684	426	450	2.60	2.00	3.33	3.00	3.00
86	6179538	725	480	95	100	3.00	2.60	3.00	2.00	2.33
87	6179255	831	940	663	573	3.00	4.00	3.33	3.67	3.67
88	6174867	106	258	166	170	2.40	2.20	3.33	2.33	2.33
89	6179457	1552	868	572	780	3.40	3.00	3.33	3.00	4.00
90	6179465	1260	840	552	340	3.00	3.00	3.00	2.33	2.67
91	6179463	852	745	375	445	3.00	3.60	2.00	2.67	3.00
92	6334696	429	1562	1091	1380	3.00	3.20	3.33	3.00	2.00
93	6336692	609	1098	1463	1427	2.80	2.40	3.00	2.00	2.00
94	6335276	169	1327	329	493	3.40	2.60	3.33	3.33	3.00
95	6418562	400	1062	709	333	3.20	2.60	4.00	3.00	3.00
96	6624543	363	3104	1423	1800	2.00	2.40	3.00	3.00	3.33
97	6624541	731	1933	1397	1920	2.60	2.40	3.00	2.00	3.00
98	6341533	483	604	1969	2680	3.20	3.40	3.67	3.33	3.33
99	6181747	1925	780	80	100	2.00	1.40	3.33	3.33	3.67
100	6181748	1456	1190	228	345	2.00	1.60	3.33	3.00	3.33
101	304660	426	1016	486	500	3.80	2.80	3.33	3.33	4.00
102	6418598	469	391	720	860	3.80	2.60	4.00	3.00	3.67
103	6418597	360	1576	311	587	4.40	3.00	4.33	3.67	3.33
104	6001673	1145	940	600	480	3.40	3.20	2.67	3.33	3.00
105	6336494	751	1207	1137	1193	3.40	4.40	2.00	3.00	3.00

106	6336482	866	1216	1774	1713	3.40	3.80	4.00	3.00	3.00
107	6342011	829	1162	1451	1787	3.40	3.80	3.67	3.00	2.00
108	6415095	663	1609	1074	1927	3.40	4.00	3.33	3.33	3.00
109	6417794	811	887	1469	1280	3.00	4.60	3.00	3.00	2.00
110	6416942	434	890	1414	1360	1.60	2.40	4.00	3.00	3.00
111	6417113	403	1049	1483	1333	3.80	3.80	4.00	3.00	3.67
112	6417117	577	2082	626	607	4.00	4.00	3.00	3.33	3.33
113	6417213	1109	1169	426	547	3.20	3.20	4.33	3.00	4.00
114	6418480	591	1680	423	387	3.60	4.00	3.67	3.00	3.33
115	6415761	1023	924	1243	1560	3.00	3.40	3.33	3.00	3.00
116	6683296	686	813	771	407	1.60	2.00	3.00	3.67	2.00
117	6681297	686	1713	674	773	3.40	3.00	1.67	2.00	3.00
118	6624336	743	1109	2054	2933	3.40	3.40	4.33	2.00	2.00
119	6418261	774	1060	1069	1080	3.60	3.20	3.67	3.00	3.33
120	6177947	79	137	221	284	2.40	2.25	3.00	3.00	3.00
121	6178556	204	318	207	215	2.00	2.00	2.67	3.33	4.00
122	6280591	88	198	132	155	3.00	3.40	4.00	3.00	3.33
123	6280415	148	345	105	172	2.40	2.60	3.33	3.67	3.33
124	6279632	236	1788	264	444	2.00	1.00	1.67	3.67	3.33
125	6279631	176	1410	108	632	1.80	1.40	2.67	2.67	2.33
126	6424403	383	651	949	2727	3.00	3.00	3.00	3.00	2.00
127	6424400	654	1387	2460	2080	3.80	3.20	3.00	1.67	1.00
128	6624413	106	376	1154	1980	4.00	2.40	3.33	2.00	2.00
129	6424865	418	660	760	470	3.40	3.00	3.67	2.67	2.33
130	6176360	903	967	2526	2900	3.00	3.20	3.00	3.00	2.00
131	6343460	440	598	857	1040	3.60	3.60	3.67	3.00	3.33
132	5435924	1196	370	692	576	3.40	3.40	3.33	3.33	4.33
133	6278943	152	135	335	300	3.00	2.00	3.33	3.00	3.67
134	6177174	756	149	293	90	2.40	2.60	4.00	3.33	3.33
135	6177148	266	675	317	655	2.40	2.60	4.33	4.00	3.67
136	6177648	209	397	718	495	3.20	4.00	3.67	2.67	3.67
137	6179222	220	1273	347	265	2.40	2.40	2.67	1.67	2.00
138	6179227	59	512	123	544	3.00	3.40	2.50	2.00	2.33
139	6624440	720	1687	1929	2593	3.40	3.20	3.00	2.33	3.00
140	6624418	509	1053	2060	2680	3.60	3.40	3.67	3.33	3.67
141	6175662	571	733	583	1813	1.60	2.00	2.33	3.33	3.33
142	6624365	534	711	820	1080	3.00	3.40	3.67	3.00	4.00
143	1987914	623	1104	2706	3047	3.40	3.40	3.00	2.00	2.00
144	72533	366	553	577	440	3.60	4.00	3.67	3.33	4.33
145	109278	1268	720	528	145	2.60	3.20	3.33	2.33	2.00
146	6178533	59	219	52	220	3.00	3.20	2.00	3.33	3.33
147	6176829	426	1260	1060	2313	2.40	1.80	2.00	3.00	3.00
148	6175076	358	337	639	727	3.20	3.20	3.50	2.00	2.00
149	6175067	843	531	1557	1767	1.80	1.60	3.33	3.67	3.00
150	6424629	291	391	2566	2187	2.60	4.00	2.00	2.00	2.00
151	6175707	156	204	634	432	3.00	3.00	2.33	2.67	3.67
152	6176368	357	420	577	700	1.40	2.40	3.33	2.00	4.00
153	6417583	494	1124	1409	1273	2.40	1.80	3.00	3.00	3.00
154	6416954	366	727	1034	840	1.60	3.00	1.67	2.00	3.00
155	6177598	275	195	116	190	2.40	2.20	3.33	3.00	4.00
156	6174884	886	1180	1460	1287	3.40	3.20	3.67	2.67	2.67

157	6174886	120	217	101	140	3.60	3.00	3.00	3.00	3.33
158	6415858	754	573	2126	2093	3.40	3.40	3.00	3.33	3.33
159	6337034	763	902	1960	2247	3.20	4.20	4.00	4.00	3.67
160	6337327	646	878	1260	1793	3.40	4.20	3.00	3.67	3.00
161	6176914	58	237	324	105	3.00	3.80	3.00	2.33	2.67
162	6176308	235	365	353	830	3.40	3.20	3.00	3.00	4.00
163	6176558	25	246	53	464	3.40	3.60	3.33	3.33	3.00
164	6179562	844	520	164	320	3.60	3.40	3.00	2.67	3.67
165	6624429	340	453	2291	2567	1.60	2.00	3.67	3.00	3.00
166	6332960	783	1800	1451	1540	3.40	3.20	3.00	2.00	2.00
167	6174892	977	1071	803	1480	3.20	3.40	3.00	4.00	3.33
168	6179347	340	240	60	310	3.20	3.40	2.00	2.67	2.00
169	6415924	469	549	1280	1280	2.40	2.40	3.33	3.67	4.00
170	6416482	597	1684	1686	1473	3.80	3.80	3.33	3.67	3.33
171	6414818	734	1107	1211	2453	2.20	2.40	3.33	3.33	3.67
172	2430154	58	262	128	465	2.40	2.60	2.33	3.33	3.00
173	6278982	640	153	510	280	2.00	1.00	4.00	3.33	3.00
174	6278849	212	155	85	80	2.00	1.60	4.00	3.00	4.33
175	6279585	128	244	120	775	3.60	3.00	4.00	3.33	3.67
176	6424861	192	180	205	95	3.20	2.40	3.33	3.33	3.33
177	6278811	256	135	195	132	1.40	1.80	3.33	3.00	4.00
178	6278812	160	345	388	480	2.40	2.20	2.33	2.00	2.00
179	3822784	232	308	120	108	3.40	3.40	2.33	2.67	4.00
180	6280583	224	105	124	80	3.40	3.20	4.33	3.33	4.00
181	6280588	124	333	80	136	3.20	3.40	4.00	3.33	3.33
182	6280393	135	170	160	75	3.00	2.80	3.00	2.67	3.00
183	6280381	95	170	85	272	2.40	2.40	3.00	2.00	4.00
184	6487731	214	982	1091	1293	3.20	3.60	3.00	3.33	3.33
185	3855011	1332	250	555	470	3.00	3.60	3.00	3.00	3.00
186	6424874	274	636	615	580	2.40	2.00	3.00	3.00	3.33
187	6278810	212	190	200	440	3.20	3.20	4.00	3.00	3.33
188	6279005	124	250	256	125	2.40	2.40	4.00	3.00	3.67
189	6487349	149	362	880	1253	2.60	3.00	3.33	3.00	3.00

Table S3. Arithmetic means of check spring wheat lines assessed under growth room (GR) conditions, greenhouse (GH), and field conditions (CR_Y and CR_K) for root-lesion nematodes (PT and PN) and crown-rot (CR).

	Pratylenchus thornei		***Pratylenchus neglectus***		***Fusarium culmorum***				
	Growth room (GR)				***Growth room (GR)***		***Greenhouse (GH)***	***Field***	
Names of check lines	PT-1	PT2	PN-1	PN-2	CR1	CR2	CR_GH	CR_Y	CR_K
2 49 (Check-Cr,Mr)	-	-	-	-	2.20	2.40	2.30	2.20	2.20
Sunco (Check-Cr,Ms)	-	-	-	-	2.50	2.50	2.30	2.20	2.50
Altay(Check-Cr,Mr)	-	-	-	-	2.50	2.50	2.80	2.50	2.30
Seri(Check-Cr,S)	-	-	-	-	3.00	3.30	3.20	3.50	3.00
Suzen(Check-Cr,S)	-	-	-	-	2.20	2.40	2.30	2.20	2.20
Croc_1/Ae.squarrosa(224)//Opata-(Check-Pt.Pn-Mr)	247	264	243	410	-	-	-	-	-
Gatcher -(Check-Pt,Pn-S)	832	670	626	660	-	-	-	-	-

Gs50a-(Check-Pt-Mr, Pn-S)	563	557	720	970	-	-	-	-	-
Seri -(Check-Pt,Pn-S)	890	944	926	1100	-	-	-	-	-
